# National Insurance and the Employees of Hospitals

**Published:** 1912-03-16

**Authors:** Harry Johnson

**Affiliations:** House Governor and Secretary. Leicester Infirmary


*To the Editor of* The Hospital.

### National Insurance and the Employees of Hospitals.

*To the Editor of* The Hospital.

Sir,—I am very pleased indeed that you are devoting pages of The Hospital to points of interest in connection with the National Insurance Act, and I hope the publicity will induce boards of hospitals to take into immediate consideration the question of the insurance of their employees.

You refer to the fact that the Board of Governors of the Leicester Infirmary had already considered the matter. They did this for three reasons :—

1. To learn themselves of their position under the Act as employers.

2. That the employees should be informed as to the advantages of the Act.

3. That the joint contributions of the board and the staff might be used to the fullest advantage of the staff.

At the time they gave consideration to the matter they were unaware of any society directly connected with the English hospitals in a position to deal with the question of insurance of hospital staffs.

Subsequently, however, the Royal National Pension Fund for Nurses has intimated the formation of the Nurses' National Insurance Society to insure trained female nurses (including, doubtless, nurses in course of training).

You have, on page 584 of your issue of March 9, referred in detail to the fact that the Nurses' National Insurance

Society, dealing, as its name implies, only with nurses, leaves untouched the large body of those engaged in hospital work—viz., on the female side, cooks, domestic servants, wardmaids, sewing-maids, laundresses, and others; on the male side, clerical staffs, dispensers, gardeners, porters, attendants, joiners, stokers, and others.

A percentage of male staffs are not at present members of Friendly Societies; the majority of mechanics will doubtless belong to Trades Unions or Friendly Societies.

Assuming, as we may, that a majority of trained nurses may prefer to join a professional society, such as the Nurses' National Insurance Society, the question arises as to whether there is a sufficiency of employed persons in hospitals, infirmaries, convalescent homes, cottage hospitals, and similar institutions, to warrant the formation of an approved society or approved societies.

This question will not long remain in doubt if the British Hospitals Association consider the advisability of forming an approved society, as one of the first points which they will desire authoritative information upon is the question of numbers.

It will be remembered that the great requirements for approved societies are :—

1. They must not be run for profit.
2. They must be governed entirely by the members.

The second of these requirements exposes the weakness of one central approved society—it must necessarily follow that administration is largely in the hands of members resident within easy reach of the offices, as members living at a distance would find it difficult to attend meetings.

A more perfect scheme appears to be to divide the whole of England into geographical areas and form approved societies for each area, combining, however, with a central association for the purposes of reserves or deficits. This would give insured persons the substantial self-government which the Act demands.

Other advantages would be :—

1. All institutions in the area could be linked together.
2. Meetings could possibly be held at the respective institutions in turn.
3. Cost of administration would be light, as doubtless some of the detail work could be performed honorarily.

You have pointed out the necessity of taking definite steps at once. You have also referred to the reserve of, roughly, 3½ million pounds to be provided each year for the first 18 years' working of the Act. This reserve will come out of the contributions of insured persons, and the fact that during these initial years such a large provision is made shows at once the possibilities of the future.

Undoubtedly a portion of this provision should, after the 18 years have expired, be allocated to approved societies in connection with the hospitals. This is an important reason for mature consideration of the whole position by an authoritative body associated with the hospitals, to say nothing of the bond of union between hospital boards and their staffs, which would be strengthened by the formation of approved societies.—I am, yours faithfully,

Harry Johnson.
House Governor and Secretary.

Leicester Infirmary, March 11.

